# New Insight into Mechanisms of Protein Adaptation to High Temperatures: A Comparative Molecular Dynamics Simulation Study of Thermophilic and Mesophilic Subtilisin-Like Serine Proteases

**DOI:** 10.3390/ijms21093128

**Published:** 2020-04-28

**Authors:** Peng Sang, Shu-Qun Liu, Li-Quan Yang

**Affiliations:** 1 College of Agriculture and Biological Science, Dali University, Dali 671000, China; pengsang@dali.edu.cn; 2 State Key Laboratory for Conservation and Utilization of Bio-Resources in Yunnan, Yunnan University, Kunming 650000, China

**Keywords:** solvent, serine proteases, molecular dynamics simulation, free energy landscape

## Abstract

In high-temperature environments, thermophilic proteins must possess enhanced thermal stability in order to maintain their normal biological functions. However, the physicochemical basis of the structural stability of thermophilic proteins at high temperatures remains elusive. In this study, we performed comparative molecular dynamics simulations on thermophilic serine protease (THM) and its homologous mesophilic counterpart (PRK). The comparative analyses of dynamic structural and geometrical properties suggested that THM adopted a more compact conformation and exhibited more intramolecular interactions and lower global flexibility than PRK, which could be in favor of its thermal stability in high-temperature environments. Comparison between protein solvent interactions and the hydrophobicity of these two forms of serine proteases showed that THM had more burial of nonpolar areas, and less protein solvent hydrogen bonds (HBs), indicating that solvent entropy maximization and mobility may play a significant role in THM’s adaption to high temperature environments. The constructed funnel-like free energy landscape (FEL) revealed that, in comparison to PRK, THM had a relatively flat and narrow free energy surface, and a lower minimum free energy level, suggesting that the thermophilic form had lower conformational diversity and flexibility. Combining the FEL theory and our simulation results, we conclude that the solvent (entropy force) plays a significant role in protein adaption at high temperatures.

## 1. Introduction

Thermophiles should have a set of adaptive mechanisms to ensure the stability (homeostasis) of their physiological functions under extreme high temperature conditions, including cell integrity, sufficient metabolic rate, and stability of protein and genetic material [1]. How to discover, describe, and explain these mechanisms is a priority in order for human beings to begin to understand extreme life forms as well as the driving force behind biodiversity [2]. Enzymes are proteins that are needed for a majority of biological activities and physiological processes within organisms. Therefore, secretion of enzymes that are capable of performing normal catalytic functions at high temperatures is a key prerequisite for thermophiles to adapt to high temperature conditions.

Temperature is the key external factor that determines protein dynamics and enzyme catalytic efficiency [3,4,5]. Increases in temperature will increase the thermal or kinetic energy of atoms, which could further lead to the increase of protein mobility and instability. Therefore, in order for thermophilic enzymes to maintain normal catalytic function under extreme high temperature conditions, thermophilic enzymes must possess a unique set of dynamic properties to ensure their structural stability and conformational flexibility [6,7]. In theory, thermophilic enzymes require higher structural rigidity and more compact conformation to ensure that they are stable enough at high temperature conditions (for example, 60 °C or higher) without thermal denaturation. Current research suggests that salt bridges provide greater stabilization to the thermophilic than mesophilic enzymes, which indicate that electrostatic interactions play a significant role in protein adaptation to high temperatures [8,9]. In addition, thermophilic enzymes tend to have more non-covalent interactions (enthalpy force) when compared with psychrophilic or mesophilic enzymes in the same family [10,11]. However, thermophilic enzymes from different families may adopt different enthalpy force factors (hydrogen bonds, vander Waals interactions, metal ion binding, aromatic ring stacking, cation-π interactions, etc.) to ensure overall structural stability at high temperatures. This indicates that non-covalent interactions may not be the determinant or physicochemical reason for enzymes’ adaptation to high temperature. In addition, several experimental and theoretical studies have shown that the extra thermal stability of thermophilic enzymes can be achieved by decreasing the conformational entropy gain associated with denaturation [12]. Accordingly, the entropy effect should be taken into account when exploring the physicochemical basis for the increased thermal stability of thermophilic enzymes apart from the electrostatic interactions and other non-covalent interactions.

The interaction between proteins and solvents plays a key role in protein folding, structural stability, flexibility and function [2,13,14]. For example, the hydrophobic effect (solvent entropy maximization or force) has been considered as the most important driving force of protein folding, and the most important factor in determining protein structural stability [15,16]. Changes in temperature will lead to changes in the physicochemical properties and mobility of the solvent, thus affecting the interaction between protein and solvent molecules [17,18]. As temperature increases, solvent entropy will increase due to the weakened hydrogen bond strength between the solvent molecules. This could further squeeze the exposed hydrophobic groups into the protein interior, thus making the stability of the protein enhanced to a certain extent [16]. Nevertheless, when the denatured temperature is reached, the extremely intense mobility of the solvent molecules will destroy all kinds of enthalpy forces that maintain the protein structure stability, which could expose the hydrophobic groups embedded in the protein interior, and eventually lead to hydrophobic group entropy increase and protein thermal denaturation [19]. Although there are only minute conformational differences between homologous thermophilic and mesophilic static structures, the difference in their amino-acid composition could cause them to have varied surface composition polarity, which would cause them to undergo different interactions with solvent molecules. Therefore, it is necessary to explore how thermophilic enzyme utilize or resist the effect of solvent entropy increase to maintain structural stability under high temperature conditions, which is an important step in starting to elucidate the adaptation mechanism of thermophilic enzymes.

At present, the protein solvent system free-energy landscape (FEL) theory provides a theoretical basis for the in-depth understanding of protein dynamics and its interactions with solvents [14,20,21]. The FEL defines two dynamic properties of the protein solvent system: the thermodynamic property (i.e., the relative probabilities of the protein conformational states) and the kinetic property (i.e., the free-energy barrier between these conformational states). Under the FEL theory, entropy change, especially entropy change of solvent molecules, is considered to be the most fundamental cause of the fluctuation of the free-energy of the system [14]. Since temperature determines the mobility/entropy of the solvent, it also plays an important role, through affecting the protein solvent interactions, in shaping the profile of the funnel-like FEL, including the width, depth, and ruggedness of the funnel. Based on the FEL theory, D’Amico et al. proposed an ideal FEL model to rationalize the stability-flexibility relationships in extremophilic enzymes’ adaptation to extreme temperatures [22]. This model shows that the FEL of thermophilic enzymes can be depicted as having a single or only a few minima with high energy barriers between them, thus making thermophilic enzymes have lower conformational flexibility and more stability, when compared to the psychrophilic and/or mesophilic enzymes. Although this model elaborates on the physicochemical basis of the extremophilic enzymes’ stability-flexibility relationships, there have not been any true, feasible FEL reported at present.

**Figure 1:**
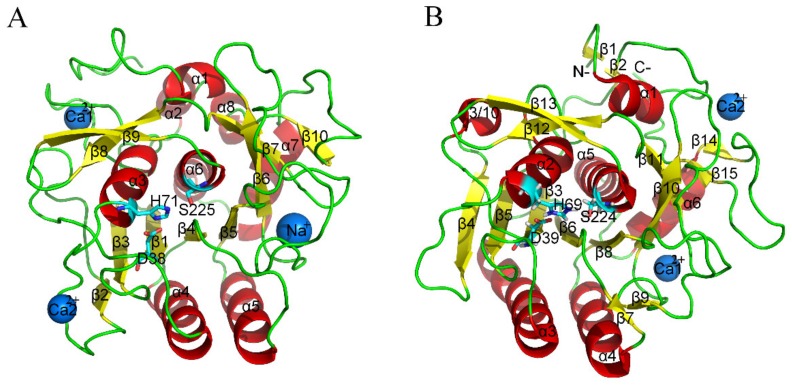
Ribbon representation of thermophilic (**A**) and mesophilic (**B**) serine proteases. These structures were obtained from PDB with PDB codes 1THM and 1IC6. α-helices, β-strands, and loops are colored red, yellow, and green, respectively. The catalytic triad residues are shown as stick models. Ca^2+^ and Na^+^ are shown as blue spheres. (**A**) and (**B**) were generated using Pymol [24].

Serine proteases are enzymes that can hydrolyze peptide bonds in proteins, and they present ubiquitously in almost all organisms [23]. Due to their ubiquity and clear catalytic mechanism, serine proteases are often deemed as excellent objects in which to study enzyme temperature adaptation. In this study, thermophilic serine protease (THM) from *Thermoactinomyces vulgaris* and its homologous mesophilic counterpart (PRK) from *Tritirachium album* (Figure 1) were used as starting structures for comparative molecular dynamics (MD) simulations. To explore the physicochemical basis for enzyme adaption at high temperatures, the dynamic structural and geometrical properties including intramolecular and protein solvent interactions as well as protein hydrophobicity were compared between these two forms of serine proteases. The FELs of THM and PRK were also constructed and compared in order to explore the mechanism underlying enzyme adaptation to high temperature.

## 2. Results

### 2.1. Trajectory Stability and Evaluation of Conformation Sampling

**Figure 2:**
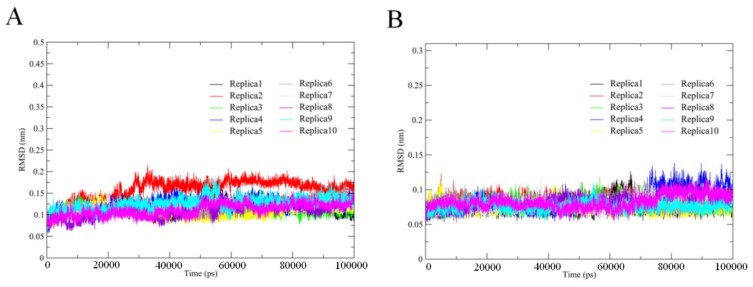
Evolutions of the backbone RMSD values of the THM and PRK with respect to their starting structures during the 10 independent MD simulations (Replicas 1–10). (**A**) THM; (**B**) PRK.

We used MD simulations to investigate and compare thermophilic and mesophilic serine proteases. In order to determine the stability/equilibrium of our simulations, we calculated the backbone root mean square deviation (RMSD) for each replica of the two simulation systems as a function of time. We observed that the backbone RMSD of each replica steadily increased for ~30 ns from the start of the simulation and then stabilized (Figure 2). Therefore, for each replica, we concatenated the MD trajectories from 30–100 ns to obtain a single 700-ns trajectory that was used for subsequent determination of the structural properties and essential dynamics (ED) analyses.

We next evaluated the convergence of our conformational sampling. We computed cosine content values from the first few eigenvectors obtained from the ED analyses of the replicas and joined trajectories (Table 1). Random diffusion protein dynamics resulted in a high cosine content value (i.e., close to 1) and indicates insufficient sampling relative to the simulation timescale. Conversely, converged sampling yielded a cosine content value close to 0. The eigenvector cosine content values were significantly higher than the corresponding single joined trajectory values for each simulation replica. These results demonstrate that a high degree of sampling convergence can be achieved by having multiple simulation replicas.

### 2.2. Flexibility Analysis

**Figure 3:**
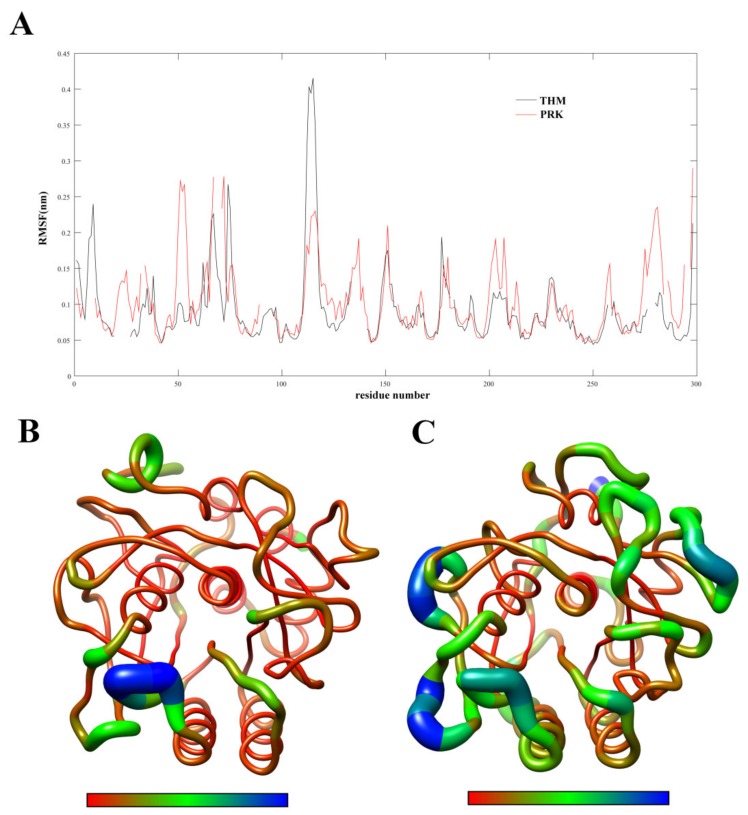
Comparison between the structural flexibility of THM and PRK. (**A**) Per-residue average backbone RMSF profiles calculated from MD trajectories of THM (black line) and PRK (red line). The gap of the curves corresponds to the insertion or deletion in the structural alignment. The residues are numbered according to the PRK structure. (**B**,**C**) are 3D backbone representations of the THM and PRK structures mapped with per-residue average backbone RMSF values, respectively. The backbone color ranges from red to blue and corresponds to a line from thin to thick and denotes that the backbone RMSF varies from the lowest to the highest values. (**B**,**C**) were generated using UCSF Chimera [25].

In order to examine the molecular flexibility of these two proteases, we computed the RMSF values of their C_α_ atoms from the joined MD trajectories. Figure 3A shows the C_α_ RMSF values as a function of residue number. Figure 3B,C are the three-dimensional (3D) backbone representations with the thickness/color of the worm corresponding to RMSF values. As shown in Figure 3, although many features are common to both proteases (e.g., the surface-exposed loops and N-, C- termini exhibit high RMSF values and the regular secondary structure elements and buried hydrophobic core exhibit low RMSF values), most structural regions are characterized by higher RMSF values in PRK compared to THM. Furthermore, the average RMSF values calculated over all the C_α_ atoms of THM and PRK are 0.81 and 0.92 Å, respectively, indicating a higher global rigidity of the thermophilic THM compared to the mesophilic PRK. The higher rigidity of THM can be expected to enhance its thermostability in high-temperature environments.

### 2.3. Geometrical and Structural Properties

For these two proteases, various geometrical and structural properties, including NHB, SASA, NSB, NNC, and Rg, were calculated based on the joined MD trajectories. Table 2 lists the average values and standard deviations of these properties. For all properties listed, the average values are very similar between the two proteases, with only minor differences observed. The subtle differences in these values can still reflect the changes in structure and stability between THM and PRK. For example, THM has more NNC, NSB and static HBs than PRK, indicating a higher number of stabilizing interactions or contacts in THM during simulation. When compared to PRK, THM has lower average values of Rg and SASA, revealing that THM is on average more tightly packed than PRK. This may also explain why THM has more static HBs and inter-atomic contacts than PRK.

We also computed the number of dynamic HBs (i.e., total number of HBs formed in a simulation) formed within the protein and between the protein and the solvent. There are 1592 and 1952 intramolecular dynamic HBs in the THM and PRK trajectories, respectively, as well as 328,834 and 331,932 protein solvent dynamic HBs in the THM and PPK trajectories, respectively. Since the dynamic HBs reflect the competitive HB interactions, a higher number means a larger freedom of competition and hence greater protein flexibility. The above results suggest that the number of dynamic HBs has been optimized to lower the conformational flexibility of THM for its adaptation to high-temperature.

### 2.4. Essential Dynamics (ED) Analyses and Free-energy Landscapes

**Figure 4:**
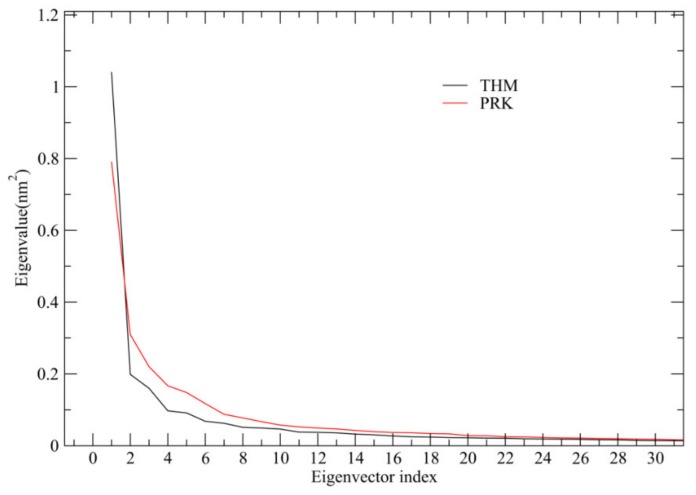
Eigenvalues as a function of the eigenvector index. Only those of the first 30 eigenvectors are shown.

After diagonalization of the covariance matrix built from the concatenated trajectories, ED provides not only a set of eigenvectors and eigenvalues but also the value of total mean square fluctuations (TMSF). For THM and PRK, the TMSF values are 3.16 and 3.59 nm^2^, respectively, suggesting that THM experienced decreased amplitude of atomic fluctuations during simulation. Thus THM exhibited a lower overall flexibility compared to PRK, in agreement with the result of the RMSF analysis. The eigenvalues as a function of the eigenvector index was plotted to evaluate the amplitude of fluctuations along eigenvectors. As shown in Figure 4, for both proteases, only in the case of the first feweigenvectors can the significantly large eigenvalues be observed. Notably, the first two eigenvectors contribute 39.2% and 30.6% of THM and PRK, respectively to the TMSF values. This indicates that these TMSF values account for a significant fraction of the overall atomic fluctuations. Therefore, the projection of the first two eigenvectors/PCs can be used to approximately characterize the collective molecular motions of THM and PRK and, consequently, distinguish amongst different conformational substates.

**Figure 5:**
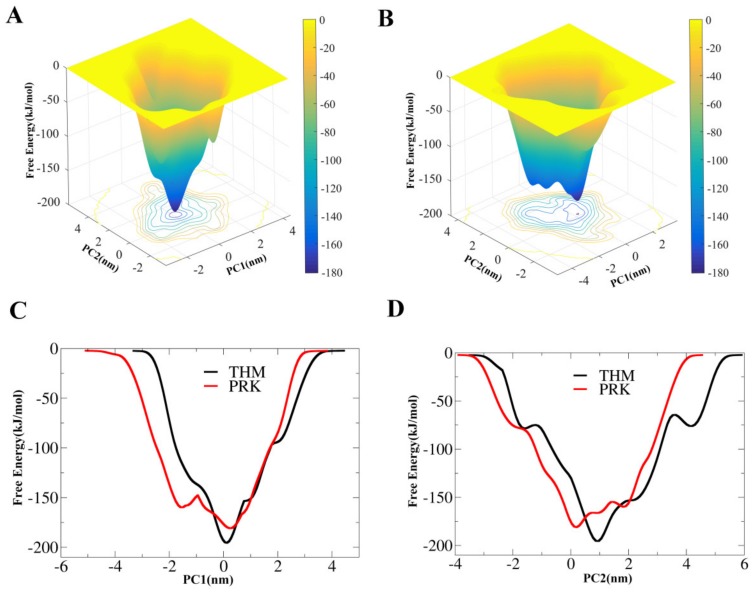
Constructed free-energy landscapes (FELs) of THM and PRK. (**A**) and (**B**) are 2D FELs for THM and PRK as a function of the first (PC1) and second (PC2) eigenvectors, respectively. The color bar represents the free-energy value in kJmol^−1^. (**C**) and (**D**) are 1D free-energy profiles for THM and PRK as a function of PC1 and PC2, respectively.

For the two proteases, their FELs were constructed based on well-tempered metadynamics simulations using the projections of the first (PC1) and second (PC2) eigenvectors as the CV1 and CV2, respectively. Figure 5A,B show the 2D FELs of THM and PRK as a function of PC1 and PC2, respectively. Although both FELs present a funnel-like shape, there is only one main free-energy well/basin at the bottom of FEL (i.e., the global free-energy minimum region) for THM but three main wells/basins at the bottom of FEL for PRK, indicating that PRK has more stable conformational substates than THM. Figure 5C,D show the 1D free-energy profiles of these two proteases along PC1 and PC2, respectively. It is clear that THM has a lower global minimum free-energy value (or deeper funnel depth) compared to PRK. Furthermore, the free-energy profiles appear to be narrower, particularly at the lower half of the funnel for THM than for PRK.

## 3. Discussion

In order to understand the structure-function relationship of thermophilic enzymes and elucidate the mechanism by which they have adapted to high-temperature, it was necessary to perform a comparative study of the dynamic properties between thermophilic enzymes and their homologous mesophilic counterparts. Further, the FELs of these two form of homologous enzymes needed to be constructed and compared in order to evaluate the influence of solvent molecules on the conformational state with regards to protein dynamics. To explore the physicochemical basis of structural stability on thermophilic enzymes at high-temperatures, we performed MD simulations on thermophilic and mesophilic subtilisin-like serine proteases. Since our previous study showed that the mesophilic PRK did not unfold below 373 K [16], we selected 340 K as the temperature for MD simulations in this study. This was because 340 K is the optimal growth temperature for *Thermoactinomyces vulgaris* when it is secreting thermophilic THM [9]. The difference in temperature selection for MD simulations was decided on in an effort to emphasize the differences between these two types of enzymes in high-temperature tolerance. Nevertheless, in order to obtain more robust conclusions, we also performed 100 ns MD simulations at 300 K for PRK and THM, respectively (Figures S1–S3, Table S1). Comparisons of the dynamic structural and geometrical properties and the results of the ED analyses in both the 340 K and 300 K simulations pointed to a common conclusion – that thermophilic THM adopted a more compact conformation and exhibited more intramolecular interactions (including electrostatic interactions) and less global flexibility compared with mesophilic PRK. This finding is corroborated by the thermophilic THM’s thermal stability in high-temperature environments.

Under the FEL theory, solvent entropy maximization (hydrophobic effect) is considered to be the driving force in protein folding, and solvent entropy maximization is a critical factor in determining protein structural stability [15]. To elucidate the physicochemical basis as well as the role that solvents play in protein adaption in high-temperature environments, we computed the hydrophobicity (defined as the fraction of the buried non-polar area vs. the total nonpolar area) of these two forms of serine proteases. For THM and PRK, the estimated hydrophobicities were 40% and 38%, respectively, indicating that the hydrophobicity of thermophilic THM is slightly stronger than that of mesophilic PRK. It is widely accepted that when the hydrophobicity of a protein is stronger, its structure is often more compacted, and it has a higher capacity for resisting heat denaturation [26,27]. This can be exemplified by THM having more intramolecular contacts (NNC, NHB), smaller Rg, and smaller total SASA compared to PRK. This reflects a more compact structural packing of THM compared to that of PRK. Therefore, our results indicate that THM’s more stable and compact packing structure is most likely to arise from its stronger hydrophobicity.

A well-balanced compromise of stability and flexibility is considered to be the central issue in the adaptation of biomolecules to extreme conditions [28]. It is widely accepted that the thermostability of a protein is often associated with its decreased flexibility (or enhanced rigidity), implying that the suppression of protein internal fluctuations and mobility could be the reason it’s able to resist heat denaturation [29,30,31]. In this study, we observed that the overall fluctuation of thermophilic THM is lower than that of its homologous counterpart, supporting the viewpoint that thermal tolerance of a protein is usually correlated with reduced conformational flexibility. According to the FEL theory, protein and solvent entropy maximization (or atom mobility) are the driving forces behind the overall fluctuation of protein [14]. In particular, solvent entropy maximization has an overwhelming effect on protein internal flexibility and mobility [4,17]. Although entropy is hard to quantify, the number of dynamic HBs can be considered representative of atom mobility and competitive interactions [32]. In our study, the number of dynamic HBs within the protein and between the protein and solvent were both computed for THM and PRK, and the corresponding values were 1592 vs. 1952 and 328,834 vs. 331,932, respectively, indicating a lower level of atom mobility and competitive interactions for the thermophilic THM compared to that of the mesophilic PRK. Therefore, it is reasonable to conclude that the observed, less favorable protein solvent interactions for THM rather than for PRK are responsible for the lower global structural flexibility of THM.

Assessment of the differences between our constructed FELs revealed that, in comparison to PRK, THM has a narrower, relatively “flat” free-energy surface, and a lower minimum free-energy level, suggesting that the thermophilic form had fewer conformational substates. Since the size of these two proteins are very similar, their difference in free-energy surface could be related to their conformational flexibility, which is determined by the intramolecular competitive interactions as mentioned above. Our results have verified the ideal FEL model proposed by D’Amico et al. [22], which rationalizes the stability-flexibility relationships in extremophilic enzymes’ adaptation to extreme temperatures.

## 4. Materials and Methods

### 4.1. Preparation of the Simulated System

The high-resolution crystal structures of the thermophilic (PDB code: 1THM [33]) and mesophilic (PDB code: 1IC6 [34]) subtilisin-like serine proteases were used as starting models for MD simulations. Before simulations, all hetero atoms and crystallographic water were removed but the metal ions were retained.

### 4.2. MD Simulations

Energy minimizations and MD simulations were performed with Amber99SB-ILDN force field [35] using GROMACS software [36]. Each structural model was centered in a dodecahedron box with a 1.0 nm minimum distance between the protein and the edge of the box. Each model was then dissolved using TIP3P water model [37] along with three additional chloride ions, which were added in order to neutralize the net charge of the system. To minimize the simulation time, we used an energy minimization algorithm with the steepest descent, and we performed two continuous 500-ps position restraint simulations of 1000 kJ/mol/nm^2^ in the NVT and NPT ensembles. Conformational sampling efficiency was confirmed using ten independent 100-ns production MD simulations. A Maxwell distribution was used to determine the initial atomic velocities of each simulation. Replica 1 to 10 refer to MD trajectories with different initial velocities obtained by the same system.

During production MD runs, the system was coupled to 340 K with a 0.1 ps constant using a v-rescale [38] thermostat, and the system pressure was maintained at 1 atm with a 0.5 ps coupling constant using a weakly coupled external pressure bath [39]. The Particle-mesh Ewald (PME) method [40] was used to calculate the electrostatic interactions using an interpolation order of 4, Fourier grid spacing of 0.135 nm, and a Coulomb radius of 1.0 nm. Lennard-Jones potentials were used to model van der Waals (VDW) interactions with a cut-off distance of 1 nm. Water molecules and non-water bonds were constrained using the SETTLE [41] and LINCS [42] algorithms, respectively. Center-of-mass motion was removed at every time step, non-bonded pairs were updated every 10 time steps, and structural coordinates were saved every 10 ps.

### 4.3. Structural and Geometrical Properties

We used the following GROMACS tools to perform structural and geometrical analyses of MD trajectories: ‘gmx rms’ to determine backbone root mean square deviation (RMSD) relative to the starting structure, ‘gmx rmsf’ to determine per-residue Cα root mean square fluctuation (RMSF), ‘gmx sasa’ to determine solvent accessible surface area (SASA), ‘gmx mindist’ to calculate the number of native contacts (NNC), ‘gmx gyrate’ to measure the radius of gyration (Rg) and ‘gmx saltbr’ to calculate the number of salt bridge (NSB). The ‘gmx hbond’ tool and the ‘Hydrogen Bonds’ plugin within VMD [43] were used to determine prtein internal HBs and protein-solvent HBs, respectively. The number of existing HBs at a given snapshot of the MD trajectory is referred to as the static HB number, while the number of HBs that have ever existed in a trajectory is referred to as the dynamic HB number. To calculate the folded state buried area, the average protein SASA during the equilibrium portion of the MD trajectory was subtracted from the SASA of an extended polypeptide chain corresponding to the ideal unfolded protein state. For a given residue X, the values of the polar, nonpolar, and total SASAs were obtained from Lins et al. [44], in which they were estimated by calculating the corresponding SASAs in the Gly–X–Gly tripeptide with extended conformation.

### 4.4. Essential Dynamics (ED)

The ED method, also known as the principal component analysis (PCA) in mathematics, is widely used to extract the largest amplitude protein motions (also called collective motions or large-scale concerted motions) from an MD trajectory. A detailed mathematical description of the ED method is given by Ref. [45] Briefly, ED analysis is mainly based on the diagonalization of the covariance matrix constructed from atomic fluctuations in a trajectory, thus obtaining a set of eigenvectors (or principal components; PCs) and corresponding eigenvalues. The eigenvector is the direction in the conformational space and describes the protein collective motion during a simulation; the eigenvalue is the mean square atomic fluctuations and describes the amplitude of fluctuations along the corresponding eigenvector. The protein motion along an eigenvector can be derived by projecting the MD trajectory onto the eigenvector.

In this study, ED analyses were performed on the concatenated trajectories of THM and PRK using the GROMACS tools gmx covar and gmx anaeig. Only the Cα atoms were included in the ED analyses.

### 4.5. Free-Energy Calculations

In order to reconstruct the FELs of the thermophilic and mesophilic serine proteases, the well-tempered metadynamics simulations [46] were performed. Using metadynamics, the addition of an external history dependent potential that acts on properly chosen degrees of freedom (also called collective variables, CV) can accelerate the detection of rare events and help the system escape from local free-energy minima [47]. In our analyses, we used well-tempered metadynamics, a newly developed variant of metadynamics which enables avoiding the exploration of unphysically high free-energy regions such that the variations in the bias potential overtime are decreased, guaranteeing a smooth simulation convergence [46]. Coupling well-tempered metadynamics with a set of CVs (i.e., PCs) determined using the ED method has been shown to efficiently reconstruct a protein’s FEL [48,49]. Here, we chose projections of trajectory onto the eigenvectors 1 (PC1) and 2 (PC2) as CV1 and CV2, respectively.

We used the following parameters for the well-tempered metadynamics simulations: 0.4 kJ/mol initial Gaussian height, added every 2 ps; 0.35 nm Gaussian width; and a bias factor of 10. The other simulation parameters and conditions were the same as those used in the standard MD simulations. The starting structure for a metadynamics simulation was the final snapshot of the joined standard MD trajectory. The well-tempered metadynamics simulations were run for 500 ns for both THM and PRK using GROMACS and Plumed [49].

## Figures and Tables

**Table 1:** Cosine content values of the first two eigenvectors calculated from the ten independent equilibrium MD trajectories (30–100 ns; Replicas 1–10) and the single 700-ns joined trajectory.

Trajectory	THM		PRK	
	eig.1	eig.2	eig.1	eig.2
1	0.7491	0.3862	0.4935	0.3572
2	0.2527	0.6482	0.1579	0.5803
3	0.5723	0.3765	0.2015	0.1097
4	0.4325	0.2513	0.8605	0.2531
5	0.1708	0.2517	0.3701	0.2017
6	0.7365	0.6804	0.4971	0.0671
7	0.0376	0.4086	0.2036	0.2471
8	0.3692	0.8974	0.1912	0.4076
9	0.6702	0.3975	0.6081	0.2992
10	0.6091	0.4839	0.3582	0.5703
joined	0.0019	0.0257	0.0309	0.0093

**Table 2:** Structural and geometrical properties (standard deviations are in parentheses) of THM and PRK during MD simulations.

Protein	NNC ^a^	SASA ^b^ (Å^2^)	NSB ^c^	Rg ^d^ (Å)	NHB ^e^	
					Stat ^f^	Dyna ^g^
THM	135,765 (1139)	10,334 (191)	14 (0.08)	16.5 (0.05)	208 (7.7)	1592
PRK	133,764 (982)	11,007 (222)	10 (0.07)	16.7 (0.05)	202 (7.6)	1952

^a^ Number of native contacts. A native contact is considered to exist if the distance between two atoms is less than 6 Å; ^b^ Total solvent accessible surface area; ^c^ Radius of gyration; ^d^ Number of salt bridges. Only salt bridges with persistence greater than 20% were counted in; ^e^ Number of corresponding HBs; ^f^ Static HB number averaged over all frames; ^g^ Dynamic HB number average over all single trajectories.

## Supplementary Materials

The following are available online at https://www.mdpi.com/1422-0067/21/9/3128/s1. **Figure S1**. Time evolutions of the backbone RMSD values of the THM (black line) and PRK (red line) with respect to their starting structures during MD simulations at 300K; **Figure S2**. 3D backbone representations of protein structures mapped with per-residue average backbone RMSF values during MD simulations at 300K. (A) THM; (B) PRK; **Figure S3**. Eigenvalues as a function of eigenvector index. Only those of the first 30 eigenvectors are shown; **Table S1**. Structural and geometrical properties (standard deviations are in parentheses) of THM and PRK during MD simulations at 300K.
