# Supplementary material for: New Insight into Mechanisms of Protein Adaptation to High Temperatures: A Comparative Molecular Dynamics Simulation Study of Thermophilic and Mesophilic Subtilisin-Like Serine Proteases
Source: Int J Mol Sci. 2020 Apr 28;21(9):3128. doi: 10.3390/ijms21093128 (PMC7247438; doi:10.3390/ijms21093128)
Supplement: Supplementary file 1 [file ijms-21-03128-s001.pdf]

Article

New Insight into Mechanisms of ProteinAdaptation to High Temperatures: A Comparative Molecular Dynamics Simulation Study of Thermophilic and Mesophilic Subtilisin-Like Serine Proteases

Peng Sang [1], Shu-Qun Liu [2,*] and Li-Quan Yang [1,*]

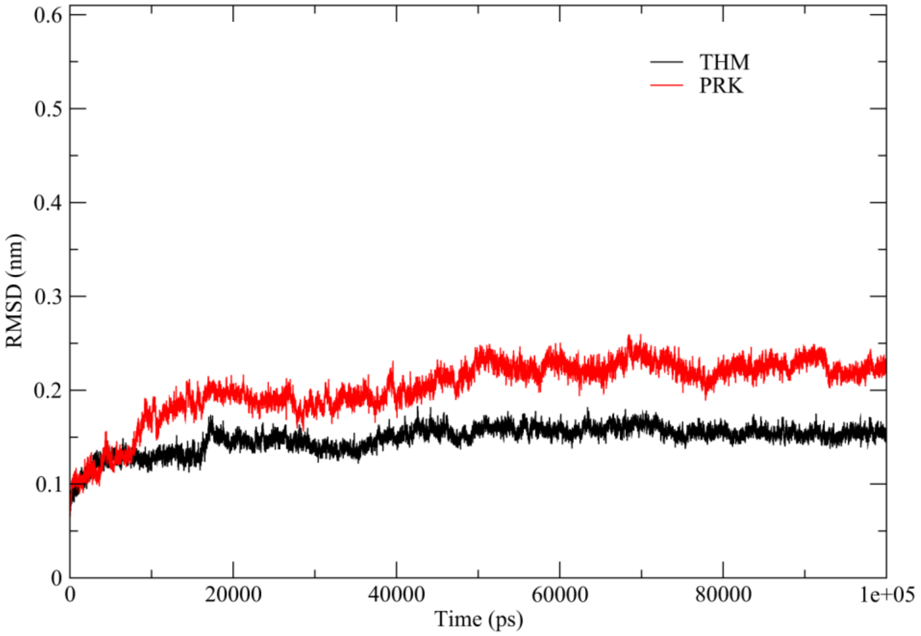

Figure S1. Time evolutions of the backbone RMSD values of the THM (black line) and PRK (red line) with respect to their starting structures during MD simulations at 300K.

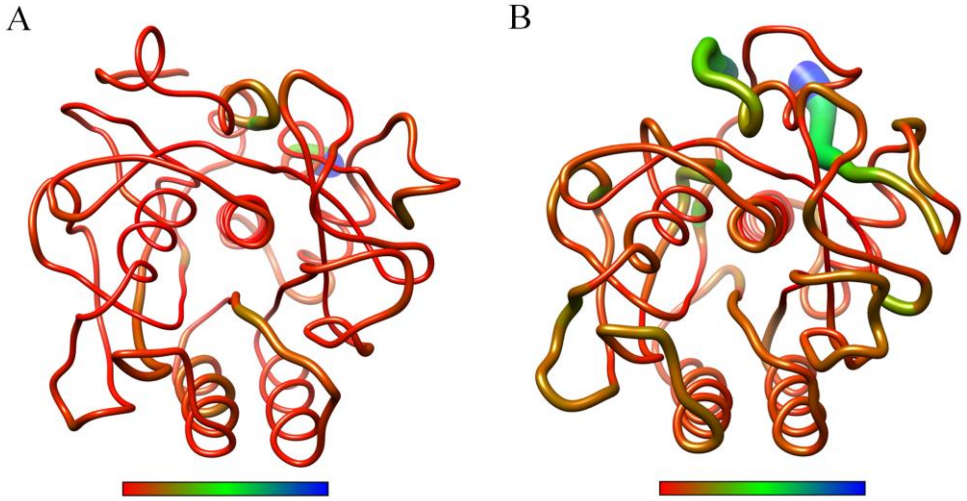

Figure S2. 3D backbone representations of protein structures mapped with per-residue average backbone RMSF values during MD simulations at 300K. (A) THM; (B) PRK.

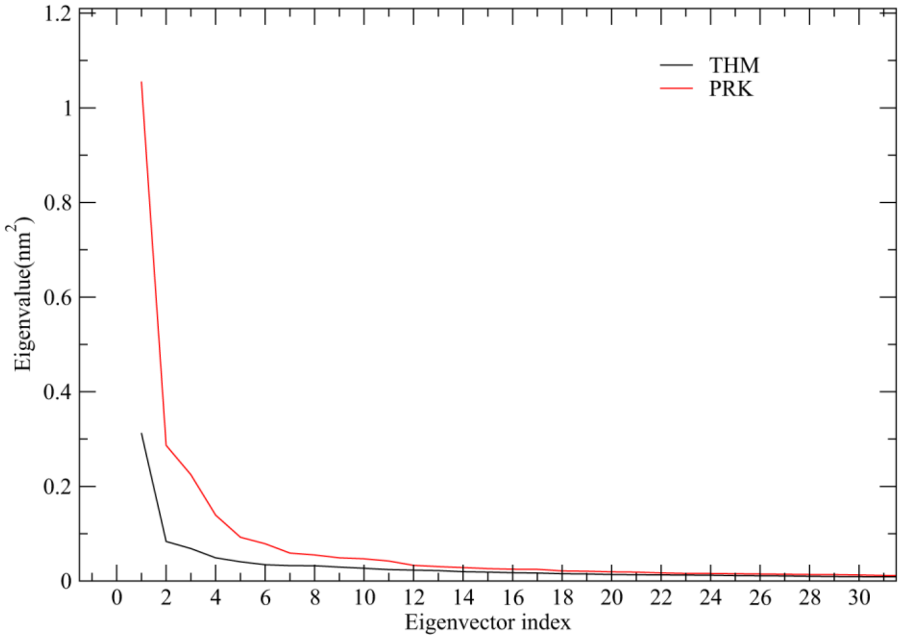

Figure S3. Eigenvalues as a function of eigenvector index. Only those of the first 30 eigenvectors are shown.

Table S1. Structural and geometrical properties (standard deviations are in parentheses) of THM and PRK during MD simulations at 300K.

Protein	NNC [a]	SASA [b] (Å²)	Rg [c] (Å)	NHB [d]	
				Stat [e]	Dyna [f]
THM	135235 (925)	10336 (157)	16.5 (0.04)	213 (7.5)	2024
PRK	133497 (803)	10916 (197)	16.6 (0.05)	198 (7.0)	2443

[a] Number of native contacts. A native contact is considered to exist if the distance between two atoms is less than 6 Å; [b] Total solvent accessible surface area; [c] Radius of gyration; [d] Number of corresponding HBs; [e] Static HB number averaged over all frames; [f] Dynamic HB number average over all single trajectories.
